# Polymorphic renal transporters and cisplatin’s toxicity in urinary bladder cancer patients: current perspectives and future directions

**DOI:** 10.1007/s12032-022-01928-0

**Published:** 2023-01-17

**Authors:** Mohamed S. Selim, Amira B. Kassem, Noha A. El-Bassiouny, Ahmad Salahuddin, Raghda Y. Abu El-Ela, Marwa Samir Hamza

**Affiliations:** 1 Clinical Pharmacy Practice Department, Faculty of Pharmacy, The British University in Egypt, Cairo, Egypt; 2 Clinical Pharmacy & Pharmacy Practice Department, Faculty of Pharmacy, Damanhour University, Damanhour, Egypt; 3 Biochemistry Department, Faculty of Pharmacy, Damanhour University, Damanhour, Egypt; 4 Medical Oncology Department, Faculty of Medicine, Fayoum University, Fayoum, Egypt; 5 Biochemistry Department, Scientific Research Center, Al-Ayen University, Thi-Qar, Iraq

**Keywords:** Urinary bladder cancer, Cisplatin, Polymorphisms, Nephrotoxicity, Transporters

## Abstract

Urinary bladder cancer (UBC) holds a potentially profound social burden and affects over 573,278 new cases annually. The disease’s primary risk factors include occupational tobacco smoke exposure and inherited genetic susceptibility. Over the past 30 years, a number of treatment modalities have emerged, including cisplatin, a platinum molecule that has demonstrated effectiveness against UBC. Nevertheless, it has severe dose-limiting side effects, such as nephrotoxicity, among others. Since intracellular accumulation of platinum anticancer drugs is necessary for cytotoxicity, decreased uptake or enhanced efflux are the root causes of platinum resistance and response failure. Evidence suggests that genetic variations in any transporter involved in the entry or efflux of platinum drugs alter their kinetics and, to a significant extent, determine patients’ responses to them. This review aims to consolidate and describe the major transporters and their polymorphic variants in relation to cisplatin-induced toxicities and resistance in UBC patients. We concluded that the efflux transporters ABCB1, ABCC2, SLC25A21, ATP7A, and the uptake transporter OCT2, as well as the organic anion uptake transporters OAT1 and OAT2, are linked to cisplatin accumulation, toxicity, and resistance in urinary bladder cancer patients. While suppressing the CTR1 gene’s expression reduced cisplatin-induced nephrotoxicity and ototoxicity, inhibiting the expression of the MATE1 and MATE2-K genes has been shown to increase cisplatin’s nephrotoxicity and resistance. The roles of ABCC5, ABCA8, ABCC10, ABCB10, ABCG1, ATP7B, ABCG2, and mitochondrial SLC25A10 in platinum-receiving urinary bladder cancer patients should be the subject of further investigation.

## Introduction

According to GLOBOCAN Estimates 2020, urinary bladder cancer (UBC) holds a significant social burden, affecting over 573,278 new cases and resulting in 212,536 fatalities [1]. Males are predominantly affected by UBC more than females, with a ratio of almost (3:1). Also, it is a disease of the elderly, with 55% of those diagnosed being over the age of 75 [2]. UBC risk factors are best classified into two categories: inherited genetic predispositions, where first-degree relatives of UBC have a two-fold increase in risk, and occupational exposures to tobacco smoking, which is regarded as the most significant risk factor for UBC, accounting for 50% of cases [3]. Painless haematuria, dysuria, irritating voiding, and pelvic soreness are all common presenting symptoms of UBC [4].

Based on histology and staging, bladder cancer is classified into two essential disease states at the time of diagnosis. The first is non-muscle invasive bladder cancer (NMIBC), which is limited to the bladder’s mucosal or submucosal inner lining. NMIBC is of low grade, accounts for approximately 70% of UBC occurrences, and demonstrates a 5-year survival rate of 90%. Even though they are not possibly life-threatening, they have a proclivity for recurrence, necessitating lifetime monitoring [5]. The second type is muscular invasive bladder cancer (MIBC), however, it is a potentially lethal form of UBC that has progressed into the bladder’s detrusor muscle lining and presumably beyond, posing a significant threat to further metastatic dissemination. Furthermore, the extent of invasion can be assessed by computed tomography or magnetic resonance imaging [6].

The management of bladder cancer is typically governed by the pathologic degree of the disease and the consequent staging using the tumor-node-metastasis classification system [7]. Intravesical Bacillus Calmette-Guerin (BCG) therapy is a promising treatment for NMIBC, and it was the first therapy to reduce the chances of recurrence and progression to high-grade NMIBC [8]. However, the current global scarcity of BCG has led to higher recurrence rates and raised the number of patients needing cystectomies [9, 10]. Transurethral resection of the tumor accompanied with intravenous chemotherapy, or BCG, is the standard of care for the initial treatment of a UBC. Following the failure of BCG therapy, radical cystectomy is usually suggested [11]. Radical cystectomy with bilateral pelvic lymph node dissection has been the hallmark therapeutic intervention for localized and locally advanced disease [12]. The bladder, seminal vesicles, prostate, and regional lymph nodes are all excised during a radical cystectomy in males. Women, on the other hand, have their bladder, urethra, vagina, uterus, distal ureters, and regional lymph nodes removed [13].

Over the past 30 years, the US Food and Drug Administration (FDA) and the European Medicines Agency have authorized some drugs for the management of NMIBC, including valrubicin, mitomycin, atezolizumab, and pembrolizumab [14, 15]. In contrast, bladder cancer that has spread to the muscles is primarily managed with radical cystectomy followed by neoadjuvant cisplatin-based chemotherapy, which helps to improve the outcomes, especially in patients who are at high risk of progression or recurrence [16, 17].

A combination of gemcitabine and cisplatin, or methotrexate, vinblastine, doxorubicin, and cisplatin, is coupled with a median survival period of roughly 14 months in patients with metastatic or unresectable bladder tumor [17–19]. Poor platinum sensitivity, initial impaired renal function, and other comorbidities set patients up to fall under the umbrella of cisplatin ineligibility, and those patients may benefit from immune checkpoint inhibitors and carboplatin-based treatment, whether a single-agent taxanes or gemcitabine or a carboplatin/gemcitabine combination (CarboGem) [20]. In certain cases, bladder preservation is a possibility, which is generally followed by chemotherapy and radiation. In individuals who are not eligible for cystectomy, radiotherapy may be employed as part of a multimodal bladder-preserving treatment or for palliation [21, 22].

Cisplatin has been shown to be effective against a wide range of solid tumors, including ovarian, testicular, bladder, colorectal, lung, and head and neck malignancies [23]. Cisplatin produces an initial response manifested in complete or partial disease remission in a number of solid tumors, including head and neck, colorectal, lung, bladder, ovarian, and testicular malignancies [23]. It is, nonetheless, associated with significant side effects such as dose-limiting nephrotoxicity, progressive peripheral sensory neuropathy, ototoxicity, and incapacitating nausea and vomiting [24]. Cisplatin’s anti-proliferative and cytotoxic actions are attributable to the drug’s propensity to attack many targets. It basically attaches to the genomic or mitochondrial DNA and creates covalent adducts with them to craft DNA lesions, prevent the creation of DNA, mRNA, and proteins, and halt DNA replication, all of which eventually lead to necrosis or death [25, 26]. Alterations in either of these events culminate in drug resistance. Cisplatin resistance mechanisms have been categorized as pre-target (those competing with cisplatin transport just before DNA binding), on-target (cellular events that occur after DNA platination), and off-target (changes in biochemical cascades that are not significantly impacted by cisplatin but interfere with its induced cell death) [27]. Since the buildup of platinum anticancer agents intracellularly is required for cytotoxicity, platinum resistance and response failure are caused by diminished influx or increased efflux [28, 29]. It has been believed for many years that platinum enters cells via passive diffusion and gated channels [30]. However, active transport mediated by multiple transporters plays an increasingly important role in platinum uptake. Therefore, three essential questions need to be addressed: which transporters are responsible for platinum absorption; how transporters change as a result of the emergence of drug resistance; and what can be done to combat resistance by focusing on transporters. [28].

As a result, genetic variations in drug transporters have a significant impact on drug kinetics and dynamics [31]. The second-generation platinum drug is carboplatin. It was initially established to mitigate the dose-limiting toxicity of cisplatin. Despite having nearly the same mode of action as cisplatin, carboplatin has a much less pronounced toxicity, as evidenced by reduced nephrotoxicity and neurological complications. Carboplatin is also appropriate for more intense high-dose chemotherapy, and it is regarded as the almost cisplatin-replacing drug in combination regimens with other treatments [32]. Oxaliplatin is a platinum drug of the third generation that was created to conquer resistance to cisplatin and carboplatin. Oxaliplatin is a platinum complex with (1, 2R)-Diaminocyclohexane and oxalate; as a bidentate oxalate, it considerably decreases oxaliplatin reactivity and so limits the harmful side effects to peripheral sensory neuropathy. Furthermore, it increases oxaliplatin’s passive uptake compared to cisplatin and carboplatin, which may explain why oxaliplatin activates different cellular invasion pathways than first- and second-generation drugs [33].

It has been evidenced that genetic alterations, particularly single-nucleotide polymorphisms, in any of the transporters involved in the uptake or efflux of platinum agents change the kinetics of these medications and, to a great extent, influence the way patients respond to them, and this influence depends on both the selected platinum agent and the tumor type [34]. Additionally, in the kidney, uptake transporters allow substances to pass from the blood into the proximal tubular cells via the basolateral membrane, whereas efflux transporters return them to the blood or urine cell via the apical membrane into luminal fluid [35]. Evidently, several of these transporter proteins are highly polymorphic. Thus, their genetic status may be a major contributor to the wide interindividual pharmacokinetic heterogeneity associated with their therapeutic usage, response, and potential toxicities [36]. Therefore, this review aims to consolidate and describe the major transporters and their polymorphic variants in relation to cisplatin-induced toxicities and resistance in urinary bladder patients.

### The organic cation transporter

#### The organic cation transporter 1 (SLC22A1)

The *SLC22A1* gene encodes the organic cation transporter 1 (OCT1), which is predominantly expressed in the liver and mediates the liver uptake of some cationic compounds [37]. Previous research ruled out cisplatin as an OCT1 or OCT2 human substrate [38], yet other studies elusively indicated that it interacts with human and rat OCT2 but not OCT1, signaling that OCT1 may exhibit a slight impact in facilitating cisplatin uptake and lethality [39]. Because human OCT1 has relatively low expression in the kidney, it is hard to ascertain its location and functional significance [40]. It has also been claimed that OCT1 is not found in human or monkey kidneys but is highly expressed in rats and mice, so it might not be of clinical importance in cisplatin uptake or resistance [41].

#### The organic cation transporter 2 (SLC22A2)

**Fig. 1:**
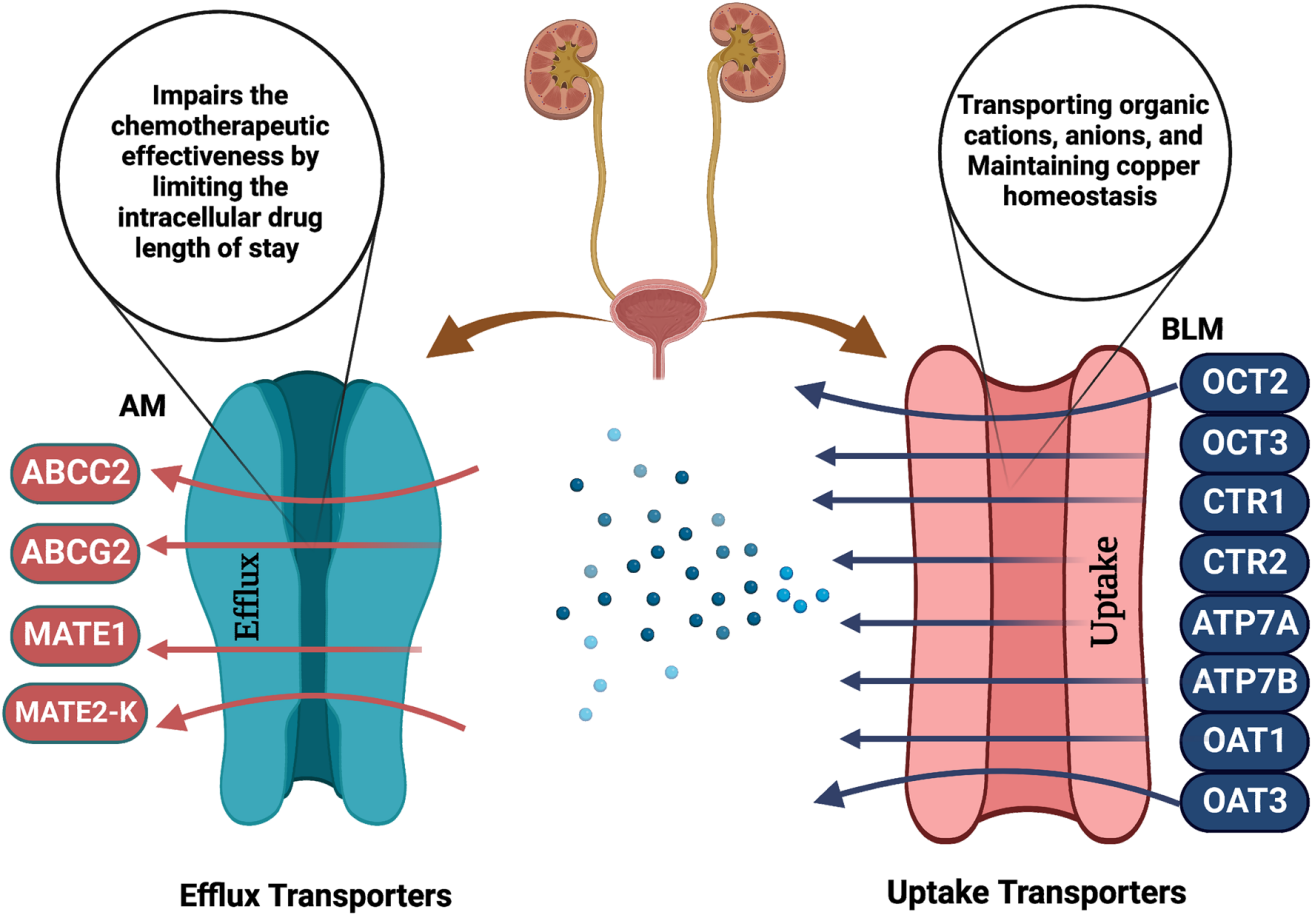
Different renal membrane sites for efflux and uptake transporter genes, *BLM*: Basolateral Membrane, *AM*: Apical Membrane

The organic cation transporter 2 (OCT2), encoded by the gene *SLC22A2*, is strongly expressed in the basolateral membrane of proximal tubule epithelial cells of the kidney and is widely thought to be a platinum compound uptake transporter (Fig. 1) [42]. Being specifically expressed in the kidneys makes them an appropriate target for research into platinum-induced nephrotoxicity protection and may as well be pivotal for the development of ototoxicity and peripheral neurotoxicity [43]. Despite the fact that nephrotoxicity may be managed with diuretics and pre-hydration therapy in the setting of UBC patients receiving cisplatin, the incidence of cisplatin nephrotoxicity remains significant [44, 45]. A previous study that was carried out on dogs revealed that the kidney retains more cisplatin than other organs [46] and that the proximal tubules are the renal structures particularly affected by cisplatin, which are also where OCT2 is expressed [46, 47]. Another study reported that patients carrying a copy of a single-nucleotide polymorphism (SNP) at the 808G > T locus (rs316019) encountered no alteration in serum creatinine following cisplatin treatment, in contrast to patients carrying the wild type, who showed indications of renal damage with a significantly elevated serum creatinine level (*P* = 0.0009). It has also been demonstrated that mice with OCT2 knockout models had dramatically decreased cisplatin renal build-up and hindered cisplatin urine excretion [48]. Four missense variants in the SLC22A2 coding area, rs316019 (Ser270Ala), were identified and linked to protective functions against cisplatin-induced ototoxicity in pediatrics (*p* = 0.022) and the adult cohort (*p* = 0.048) [48, 49], see Table 1. Furthermore, in a previous study, a SNP in OCT2 (rs316019) was found to be strongly related to platinum-induced hepatotoxicity (*P* = 0.026) and hematological toxicity (*P* = 0.039) [50], see Table 1. The first explicit in vitro evidence for cisplatin transport by human OCT2 was reported in transfected hOCT2 embryonic kidney (HEK293) cells. hOCT2 expression was likewise connected to increased cisplatin accumulation when compared to wild-type HEK293 cells [51]. In OCT2-overexpressing HEK293 cells, wedelolactone, a natural compound that selectively inhibits OCT2, was shown to reduce nephrotoxicity caused by cisplatin therapy through OCT2 suppression [52]. Carvedilol’s selective suppression of OCT1 and OCT2 shows a tendency for protection against cisplatin-induced nephrotoxicity by limiting cisplatin’s cellular entrance via OCTs [53]. A study reported that rat rOCT2 boosted cisplatin sensitivity in HEK-rOCT2-transfected cells and promoted cisplatin sensitivity [54].

**Table 1:** Examples of different SNPs and their association with the clinical outcomes of platinum chemotherapy

SNP	Gene	Subjects/Drug	Outcomes	References
rs316019	SLC22A2/OCT2	Mice/ Cisplatin	Patients carrying a copy of the SNP encountered no alteration in serum creatinine following cisplatin treatment, in contrast to patients carrying the wild type, who showed indication of renal damage with a significant elevated serum creatinine level (*P* = 0.0009)	[48]
		Human	Four SLC22A2 missense variants in rs316019 (Ser270Ala), were identified and linked to protective functions against cisplatin-induced ototoxicity in pediatrics (*p* = 0.022) and the adult cohort (*p* = 0.048)	[48, 49]
		Human	OCT2 (rs316019) was associated with hepatotoxicity (*P* = 0.026) and hematological toxicity (*P* = 0.039)	[50]
rs596881	SLC22A2/OCT2	Human/ Cisplatin	OCT2 (rs596881) has been shown to be associated with reduced risk of kidney damage brought on by cisplatin and preserved functions in terms of estimated glomerular filtration rate maintenance (*P* = 0.01)	[55]
rs420038	SLC22A3/OCT3	Human	OCT3 (rs420038) may confer a critical role in lowering colorectal cancer risk by inhibiting the SLC22A3 expression (*p* = 0.007)	[58]
rs1128503 rs2032582	ABCB1/ MRP1	Human	In urinary bladder cancer, the effects of ABCB1 SNP (rs1128503) were linked to a prolonged sirolimus half-life (*p* = 0.010) and sustained drug exposure (*p* = 0.037). A SNP (rs2032582) was also associated with a longer sirolimus half-life (*p* = 0.033)	[88]
rs12762549	ABCC2/ MRP2	Human	A SNP (rs12762549) in ABCC2 was markedly associated with severe neutropenia in patients receiving docetaxel, cisplatin, and 5-fluorouracil treatment in esophageal cancer patients (*p* = 0.014)	[98]
rs717620	ABCC2/ MRP2	Human	SNP holders had a superior response to platinum-based treatment (*P* = 0.016)	[50]
rs2273697	BCRP/ABCG2	Rats	Variant holders were shown to be associated with prolonged progression-free survival rates in advanced ovarian cancer patients receiving a combination of platinum and taxanes chemotherapy (*p* = 0.041)	[104]
rs2725264 rs4148149	BCRP/ABCG2	Human	SNP (rs2725264) and (rs4148149) in ABCG2 holders were associated with overall survival in resistant Non-small cell lung cancer patients in platinum-based combinations with a (*P* = 0.018), (*P* = 0.014) respectively	[125]
rs2289669	MATE1/ SLC47A1	Human	SNP carrier was linked to increased hematological toxicity caused by platinum treatment (*P* = 0.016)	[50]
rs10981694	CTR1/ SLC31A1	Human	SNP (rs10981694) carriers showed more susceptibility to cisplatin-induced ototoxicity in non-small cell lung cancer patients (*p* = 0.01)	[143]
rs7851395 rs12686377	CTR1/ SLC31A1	Human	rs7851395 and rs12686377 Variants holders promoted resistance to platinum therapy in non-small cell lung carcinoma in the Chinese population (*P* < 0.05)	[144]

Moreover, the *SLC22A2*/OCT2 polymorphism rs596881 has been shown to be associated with kidney protection against cisplatin-induced nephrotoxicity and preserved functions in terms of estimated glomerular filtration rate maintenance (*p* = 0.01) [55], as illustrated in Table 1.

#### The organic cation transporter 3 (SLC22A3)

The gene *SLC22A3* encodes the human organic cation transporter 3 (OCT3), and its mRNA has been detected in the placenta, liver, kidney, gut, and brain [56]. The cytotoxic impact of cisplatin was amplified by *SLC22A3* overexpression in a study assessing the influence of *SLC22A3* gene expression on the survival of patients with head and neck squamous cell cancer, in which both early as well as advanced tumor patients with elevated *SLC22A3* expression had enhanced survival rates following cisplatin treatment [57]. SNP rs420038 in the *SLC22A3* gene has been shown to inhibit *SLC22A3* expression in the Chinese population. *SLC22A3* expression was found to be greater in colorectal cancer when compared to normal tissues and thus may confer a critical role in lowering colorectal cancer risk (*p* = 0.007) [58], as shown in Table 1. The functional significance of genetic variations on the *SLC22A3* gene has not been fully understood and requires additional investigation.

#### The organic cation transporter 6 (SLC22A16)

The gene *SLC22A16* encodes the human organic cation transporter 6 (OCT6). OCT6 is one of the organic cation influx mediators [59]. An earlier study found that cisplatin uptake was enhanced in lung cancer cell lines with higher OCT6 expression and that resistance to cisplatin was directly influenced by down-regulating the transporter’s activity [60]. OCT6 overexpression in parental lung adenocarcinoma cell lines resulted in an intracellular increase in oxaliplatin concentration, indicating that OCT6 mediates platinum drug entry in cancer cells and confers resistance to them when its expression is curtailed [61]. Despite the fact that *SLC22A16* has received a considerable amount of attention with respect to platinum accumulation and resistance in various types of cancer [62, 63], no studies have explained its involvement in urinary bladder cancer, which calls for more research investigating its significance in platinum-treated UBC patients.

### The organic anion transporter

#### OAT1

The organic anion transporter 1 (OAT1), which is encoded by the *SLC22A6* gene, is one of the most abundantly expressed genes in human kidneys and is situated in the basolateral membrane, facilitating the uptake of organic anion compounds [64], as shown in Fig. 1. Many studies have demonstrated the significance of OAT1 in renal diseases such as acute renal failure and ureteral occlusion [65, 66]. Furthermore, cisplatin-induced kidney damage was significantly reduced in OAT1 knockout mice [67, 68]. A prior study demonstrated that by inhibiting OAT1 expression in rats, nanoellagic acid has managed to reverse kidney damage, oxidative stress markers, and renal tissue atrophy caused by cisplatin [69]. Conversely, a study revealed that rats that received the antioxidant lycopene in conjunction with cisplatin had significantly higher levels of OAT1 expression than rats given cisplatin alone, which in turn resulted in improved renal function. These findings suggest that lycopene can mitigate the transporter’s downregulation caused by cisplatin and that OAT1 high expression can, contrary to what was expected, enhance renal functions [70]. However, a previous investigation found that OAT1 and OAT3-deficient mice were largely shielded against cisplatin-induced nephrotoxicity. Furthermore, nilotinib, an FDA-approved treatment for some types of leukemia, has been identified as a powerful inhibitor of the organic anion transporters, and using nilotinib in conjunction with cisplatin prevents renal tubular injury [67].

#### OAT2

The SLC22A7 gene encodes the organic anion transporter 2 (OAT2) and is distinctive to the sinusoidal membrane of hepatocytes [71]. After Cisplatin therapy, the mRNA levels of the mouse kidney’s OAT1 and OAT2 were down-regulated, which helped to reduce the strain on proximal tubule cells that strive to repair damage [72]. A study reported that OAT2 is highly expressed in Hep G2, particularly in patients with hepatocellular carcinoma treated with platinum compounds, pointing out that the considerable reduction in toxicity when an OAT2 inhibitor is used implies that it may be implicated in platinum uptake via orotic acid, a well-recognized by-product of the pyrimidine biosynthesis pathway in hepatocytes [73].

#### OAT3

OAT3 *(SLC22A8)* is an organic anion drug transporter found in renal proximal tubule cells as well as brain endothelial cells [74]. According to one study, reducing the expression of the uptake transporters OAT1 and OAT3 in rats may provide preventive responses against kidney damage caused by cisplatin and uremic toxin accumulation [75]. However, in an animal study assessing Thymoquinone’s regulatory capacity on renal organic anion transporters in cisplatin-induced nephrotoxicity, where cisplatin inhibited the expression of OAT1, OAT3, Thymoquinone reversed the suppressed expression of OAT1 and OAT3 when co-administered with cisplatin, implying that Thymoquinone may offer protection against kidney injury caused cisplatin’s upregulation of the transporter [76]. In line with the previous study, successive research has been conducted and revealed that cisplatin administration markedly lowered the expression of OAT1 and OAT3 in experimental rats. For example, it was reported that curcumin difluorinated restored the expression of OATs to overcome cisplatin-related nephrotoxicity [77]. Another example is Cordyceps cicadae Mycelia, a nephroprotective Chinese medicine that has been shown to reduce cisplatin-induced kidney injury and that the addition of the Mycelia extract to cisplatin therapy markedly increased the expression of OAT1 and OAT3 in mice, confirming cisplatin’s inhibitory effect on the expression of both OAT1 and OAT3, which flares the kidney damage [78].

### The human ATP-binding cassette (ABC)

The ATP-binding cassette efflux transporters are now roughly 50 known members of the human ABC family, which is grouped into seven subfamilies, from A to G. Multidrug resistance 1 (MDR1/ABCB1), multidrug resistance protein 2 (MRP2/ABCC2), and breast cancer-related protein (BCRP/ABCG2) have received the most attention in terms of xenobiotic interactions [79, 80] as shown in (Fig. 1). Drug efflux is a key contributor to drug resistance and is believed to be controlled by the ATP-binding cassette (ABC) transporter family [81].

#### The ABCB1 gene (MDR, PGE1, and MRP1)

MDR1, which is encoded by the *ABCB1* gene, is a transport protein that belongs to the ATP-binding cassette (ABC) superfamily. It is incorporated into the cellular membranes and impairs chemotherapeutic effectiveness by limiting the intracellular length of stay of xenobiotics [82]. In a study attempting to resensitize T24R2 cisplatin-resistant bladder cancer cells to the drug via MDR1 inhibition, it was discovered that inhibiting Ets1 activity, which is an important transcription factor controlling MDR1 transcription as well as increasing chemotherapy resistance, resuscitated cisplatin activity in T24R2 cells [83]. A recent study revealed that suppressing *ABCB1* improved the susceptibility of bladder cancer cells to cisplatin by reducing cytotoxic effects and promoting cell death in UBC cells treated with cisplatin [84]. As pointed out by Hoffmann et al., increased MDR1 gene expression was tied to a poor prognosis following cisplatin as adjuvant chemotherapy in locally advanced bladder cancer, and this data might be utilized to better tackle resistance in MDR1-highly expressed patients [85]. Emodin, a multifunctional natural Chinese medicine recognized for its anti-inflammatory, anticancer, and chemoprotective characteristics [86], enhanced cisplatin cytotoxicity against T24 and J82 human bladder cancer cells via downregulating the MRP1 gene in a previous study [87]. In urinary bladder cancer, the effects of the *ABCB1* rs1128503 polymorphism on temsirolimus and its active metabolite sirolimus following administration were associated with an increased drug concentration (*p* = 0.037) and a prolonged sirolimus half-life (*p* = 0.010). Moreover, an *ABCB1* SNP (rs2032582) was also associated with a longer sirolimus half-life (*p* = 0.033) (Table 1) [88]. In a prior study, the overexpression of the *ABCB1* gene, on the other hand, coincided with elevated cisplatin resistance, hindered cytotoxicity, and overall treatment resistance in bladder cancer patients [89]. Direct suppression of the *ABCB1* gene by miR-3682-3p contributed to cisplatin and gemcitabine resistance in patients with urinary bladder cancer [90]. However, the specificity of the variability remains quite questionable owing to the cancer multidrug treatments, which propose that these combinations may not be equally carried by the same transporter [91].

#### The multi-drug-resistant protein 2 (MRP2/ABCC2 gene)

The *ABCC2* gene encodes the multi-drug resistant protein 2 (MRP2), which is a major ABC efflux transporter member that is abundantly expressed in hepatocytes as well as the apical membrane of the human proximal tubule of the kidney [92], as illustrated in Fig. 1. The *ABCG2* gene, a member of the ABC family, has been identified as a probable drug resistance gene [93]. A previous study investigated the role of forehead box protein M1, a human protein encoded by the FOXM1 gene, in predicting and influencing the frequent recurrence of non-muscle-invasive bladder cancer [94]. A successive in vitro study concluded that FOXM1 directly binds to the *ABCG2* promoter to regulate its transcription, suggesting that lowering FOXM1 expression in NMIBC patients may offer an effective response to chemotherapy and reduce the chance of BC recurrence [95]. Along with mutations in the MRP2 gene that result in a complete lack of function, numerous MRP2 SNPs with enigmatic significance have been investigated [96, 97]. Prior research found that rs12762549 in *ABCC2* (9383C > G) was markedly associated with severe neutropenia in patients receiving docetaxel, cisplatin, and 5-fluorouracil treatment [98], as shown in Table 1. In a previous study, an allele holders of *ABCC2* rs717620 SNPs had a superior response to platinum-based treatment (*P* = 0.016) [50] as listed in Table 1. Another study found that curcumin and resveratrol resensitized a human bladder cancer cell line, T24, as well as GCB-resistant cells (T24GCB) to GCB by increasing *ABCC2* expression [99]. On the other end of the spectrum, increased *ABCC2* expression has been shown to be tied to platinum resistance [100, 101]. It was concluded that suppressing the expression of *ABCC2* is likely to revive cisplatin sensitivity in platinum-resistant non-small cell lung cancer patients [102]. Moreover, in a study evaluating the seven commonest SNPs, cisplatin effectiveness, clearance, nephrotoxicity grade, progression, and overall survival showed no significance in individuals who carried neither *ABCC2 nor ABCC4* SNPs [103]. Furthermore, the *ABCC2* rs2273697 variant was linked to longer progression-free survival rates in advanced ovarian cancer patients receiving a combination of platinum and taxanes chemotherapy (*p* = 0.041) [104]; see Table 1. In a prior study, cisplatin-resistant melanoma cells displayed a distinguishable elevated expression of (MRP2/ABCC2), which was reflected by a decrease in cisplatin cytotoxicity in terms of a decline in the drug’s ability to form intra-strand DNA cross-links. This finding suggests that the suppression of the transporter may be a potential tool in mitigating resistance to platinum-based therapy [105]. MRP2 overexpression was associated in a prior study with cisplatin resistance and a poor clinical outcome in small cell lung cancer [28, 106]. Having said that, there is some evidence confirming that cisplatin is a substrate for and a reason for elevating the expression of both the *ABCC2* and *ABCC5* transporters [93, 107].

#### The multi-drug-resistant protein 5 (MRP5/ABCC5 gene)

The multi-drug-resistant protein 5 (MRP5) is encoded by the *ABCC5 gene* and is detected across most of the human organs, with exceptionally high amounts observed in skeletal muscle, the brain, and the heart [108]. [93] MRP5 expression levels were significantly higher in tissues from individuals who had received cisplatin therapy compared to those who had not in a study evaluating the in vivo expression of the *ABCC5* gene in three human lung adenocarcinoma cell lines [109]. A previous study indicated that increased expression of several multi-drug resistant protein family members, including MRP2 and MRP5, diminished cisplatin’s intracellular accumulation and potentiated the resistance of hepatocellular carcinoma cells to cisplatin and that the combined effect of glycyrrhizin and lamivudine inhibited the efflux of cisplatin out of these cell lines [110]. Taking together the available data, research proving the function of MRP5 in urinary bladder cancer patients receiving platinum treatment is still required.

#### The multi-drug-resistant protein 7 (MRP7/ABCC10 gene)

Although MRP7/ABCC10 has received a lot of attention in connection with platinum accumulation and resistance in cancers of different types [111–114], more research is required on MRP7 activity in platinum-receiving urinary bladder cancer cells.

#### The ATP-binding cassette A member 8 (*ABCA8)*

The *ABCA8* gene was found to be downregulated in a study demonstrating changes in the ABC superfamily expression levels of several antineoplastic agents, including cisplatin, which is quite contradictory to the expected overexpression of these efflux transporters. Hence, its role in drug resistance is still elusive and needs further investigation [115].

#### The ATP-binding cassette B member 10 *(ABCB10)*

[116, 118] *ABCB10* was overexpressed in non-small-cell lung cancer (NSCLC) cell lines, and downregulating it hindered NSCLC cell growth and migration [116]. Another previous study also discovered that knocking circular *ABCB10* down boosted the susceptibility of lung cancer cells to cisplatin, implying that targeting circular *ABCB10* could be a promising target for enhancing cisplatin’s effectiveness in lung cancer [117]. To date, no published work has linked the *ABCB10* gene to the status of platinum-treated UBC patients, leaving a gap to be filled by future research.

#### The ATP-binding cassette G member 1 (ABCG1)

In lung adenocarcinomas, *ABCG1* could have chemoresistance-related properties, and the depletion of ABCG1 increased the disease’s susceptibility to cisplatin therapy [118]. There has been no research to date depicting the influence of *ABCG1* variability on cisplatin-treated urinary bladder cancer.

#### The breast cancer-related protein (BCRP/ABCG2)

The breast cancer resistance protein (BCRP), also known as mitoxantrone resistance protein (MXR), is encoded by the *ABCG2* gene. As an efflux transporter, it is thought to be involved in the excretion of toxins and xenobiotics [119]. A recent study showed that downregulating the BCRP’s expression plays an essential role in effluxing folates out of the cell, which the rapidly dividing cancer cells need to properly replicate and divide. This could be associated with pathogenesis and the response of bladder cancer cells to antineoplastic therapy, but more research is needed [120]. As firmly known, Cyclooxygenase 2 (COX-2) is induced in response to inflammation [121]. Its expression in the lower urinary tract is linked to an advanced tumor grade and somehow predicts the progression of the disease [122, 123]. In a previous study, *ABCG2* overexpression was linked to poor mitomycin C (MMC) responses in bladder cancer patients. Combining a selective COX2 inhibitor, namely celecoxib, a substrate for the BCRP transporter, with MMC has strengthened the cytotoxicity of MMC in NMIBC patients, probably through a direct interaction between celecoxib and the BCRP transporter [124]. A study revealed that SNPs in the ABCG2 gene, rs2725264 and rs4148149, in platinum-based combinations were independently associated with overall survival in resistant non-small cell lung cancer patients with a (*P* = 0.018), (*P* = 0.014), respectively [125], see Table 1. In a previous study assessing the impact of the BCRP transporter on the clinical outcomes of advanced non-small cell lung cancer in a regimen incorporating cisplatin, in comparison to the BCRP-negative patients, the response rate to the treatment was lower in BCRP-positive tumors (*P* = 0.08). They also exhibited poorer rates of progression-free survival (*P* = 0.0003) and overall survival (*P* = 0.004). These results suggest that BCRP may function as an efflux transporter for a variety of anticancer agents, including platinum compounds, and that it could serve as a target for lowering chemotherapy resistance in NSCLC patients [126]. However, it should be noted that there is no proof that *ABCG2* genetic alterations would have comparable effects on human tumors as most of the available data comes from in vitro investigations. Thus, there is a need for more research using human cell lines [91].

### Mitochondrial carrier genes

#### SLC25A21

Adding to the discourse of how significant efflux transporters may contribute to reactivity and response to chemotherapy, a recent study demonstrated that the overexpression of the mitochondrial carrier SLC25A21, pertaining to the solute carrier superfamily 25, halted the growth, migration, and invasion of bladder cancer (BC) cells in vitro and suppressed the growth of the BC cells in an in vivo nude mouse model. Furthermore, in vitro, SLC25A21 knockdown promoted BC cell proliferation while inhibiting cell apoptosis [127]. Conversely, it has been observed that *SLC25A21-AS1* expression was reported to be upregulated in cisplatin-resistant nasopharyngeal cells (NPC) and that *SLC25A21* knockdown showed more effective tumor size and weight suppression with cisplatin treatment, implying that bringing down *SLC25A21-AS1* inhibited NPC cellular proliferation and multi-drug resistance in vitro and in vivo [128].

#### SLC25A10

*SLC25A10*, a mitochondrial SLC25 family member that encodes the dicarboxylate transporter, is responsible for substrate exchange in and out of the mitochondria [129]. It has been investigated as a potential target for cell metabolism and growth reprogramming. Since its knockdown made lung cancer epithelial cells A549 more susceptible to cisplatin [130], it may serve as a promising platinum sensitizer in different types of malignancies, but more research is necessary, particularly with regard to urinary bladder cancer cells.

### The multidrug and toxin extrusion 1 (MATE 1/ SLC47A1)

The *SLC47A1* gene encodes the multidrug and toxin extrusion 1 (MATE1) transporter, which is expressed in the liver as well as the brush-border membrane of the kidney’s proximal tubules [131]. It plays an important role in the efflux of a variety of cationic compounds [132]. A previous study found that MATE1 knockout mice receiving cisplatin had higher nephrotoxicity, plasma levels, and renal accumulation when compared to the wild-type, and that the expression of MATE1 in HEK293 cells enhanced the cellular uptake of cisplatin in vitro testing [133]. Despite having previously been proven to result in reduced function, only a few studies to date have identified a single-nucleotide polymorphism correlating MATE1 variations with cisplatin-induced toxicity. However, they found no link between the rs2289669G > A in MATE1 and cisplatin-induced deleterious effects. The caveat is that the two MATE isoforms (MATE2-K and MATE2-B) were not included to entirely eliminate the association [134], as shown in Table 1. In addition, the MATE1 SNP (rs2289669) was linked to hematological toxicity caused by platinum treatment (*P* = 0.016) [50]. See Table 1, a previous study reported a downregulation in the MATE-1 gene following the administration of cisplatin. The deletion of the peroxisome proliferator-activated receptor alpha (PPAR), a transcription that is abundantly expressed in the kidneys of mice, regained transporter expression and subsequently helped attenuate the cisplatin-induced nephrotoxicity [135]. The probability of developing renal damage was enhanced in research where MATE1 expression was suppressed by advanced glycation end-products in diabetic individuals [136].

### The multidrug and toxin extrusion 2-K (MATE2-K)

In mice, ondansetron boosted cisplatin-induced nephrotoxicity by inhibiting both human MATE1 and MATE2-K and mouse MATE1 in a previous study [137]. Pazopanib, an OCT2, MATE1, and MATE2-K renal transporter inhibitor, was noticed to limit the uptake of cisplatin and thus attenuate cisplatin-induced cytotoxicity in an in vitro study [138]. Drugs that suppress MATE1 and/or MATE2-K expression may intensify the intracellular accumulation of cisplatin and accelerate the drug’s induced nephrotoxicity [139, 140].

### The copper transporter 1 (CTR1)

Copper transporter 1 (CTR1), which is encoded by the gene *SLC31A1*, is essential for maintaining copper homeostasis in the cell. A yeast and mouse genetic observation study has demonstrated that the high-affinity Ctr1 holds a pivotal role in cisplatin uptake and resistance. Based on the results obtained, CTR1 activity can be preferentially enhanced in cisplatin-resistant neoplasms. Moreover, by inhibiting the expression of CTR1 in normal cells, it may be feasible to minimize cisplatin-induced nephrotoxicity and ototoxicity [141]. A comparative pre- and post-chemotherapy study of cisplatin-treated patients revealed that elevated CTR1 expression was associated with pathological downgrading in NMIBC. Admittedly, the numbers were insufficient to draw any significant conclusions [142]. CTR1 knockdown has been shown to lower cisplatin uptake by over 80% in both yeast and mouse embryonic fibroblasts [37]. A study indicated that CTR1 rs10981694 SNP carriers showed more susceptibility to cisplatin-induced ototoxicity in non-small cell lung cancer patients (*p* = 0.01) [143], as listed in Table 1. In a previous study, CTR1 rs7851395 and rs12686377 variants promoted resistance to platinum therapy in non-small cell lung carcinoma in the Chinese population. Hence, CTR1 can be used as a predictive marker for pretreatment assessment in these patients (*P* < 0.05) (Table 1) [144]. While CTR1 mediates copper uptake into the cell, it is cleared out by two *P*-type ATPases, namely ATP7A and ATP7B [36].

### ATP7A

When bladder cancer cell lines RT4 and T24 were treated with large doses of cisplatin, both ATP7A and ATP7B expression levels were significantly reduced, while CTR1 gene activity remained unaltered. This implies that bladder cancer cells may be more susceptible to drug efflux modulation than uptake [145]. An association was established between survivability and ATP7A expression, where high expression indicated poor survival in ovarian cancer patients treated with platinum-based chemotherapy, implying that increased ATP7A expression does confer a certain level of resistance to cisplatin-treated tumors [146]. Cisplatin resistance and angiogenesis were reduced by inhibiting ATP7A gene expression in esophageal cancer cells [147]. According to a study, ATP7A appears to be expressed in the majority of muscle-invasive bladder cancer patients, especially those that are resistant to cisplatin therapy. By suppressing ATP7A expression, disulfiram co-administration boosted cisplatin intracellular accumulation [148].

### ATP7B

mRNA analyses have revealed that several tumor types with elevated ATP7B expression have had a poor response to platinum therapy [149, 150]. In non-small-cell lung cancer xenograft models, increased ATP7B expression has also been identified as a sign of cisplatin resistance [151]. Additionally, studies on the potential role of ATP7B in colorectal and ovarian cancer have confirmed that its overexpression is tied to a poor response to platinum treatment [152, 153], but not in urinary bladder cancer patients, calling for further needed research to be conducted.

## Conclusion

In the current review, we aimed to cast light on the influence genetic polymorphisms can have on a variety of renal uptake and efflux transporters as well as their consequent impact on cisplatin’s response, accumulation, and toxicity in urinary bladder cancer patients. After compiling the data, we would like to first point out that suppressing the expression of the efflux transporters ABCB1, ABCC2, SLC25A21, and ATP7A has been linked to cisplatin’s accumulation and reduced resistance in urinary bladder cancer patients. Second, inhibiting the expression of the uptake transporter OCT2 has reduced cisplatin’s induced nephrotoxicity. Additionally, a reduction in cisplatin’s cytotoxicity, accumulation, survival, and increased resistance has occurred in colorectal, head, and neck cancers when OCT3 activity was suppressed, and in lung cancer when OCT6 expression was restricted. Regarding the organic anion uptake transporters, we found that downregulating the expression of both OAT1 and OAT2 reduced cisplatin-induced toxicities and safeguarded the kidneys. However, cisplatin’s toxicity was observed to be diminished, and the kidney’s structural integrity remained protected when OCT3 expression was restored. While suppressing the CTR1 gene’s expression reduced cisplatin-induced nephrotoxicity and ototoxicity, inhibiting the expression of the MATE1 and MATE2-K genes has been shown to increase cisplatin’s nephrotoxicity and resistance. Although curtailing the expression of ABCC5, ABCA8, ABCC10, ABCB10, ABCG1, ATP7B, ABCG2, and the mitochondrial SLC25A10 has enhanced cisplatin’s susceptibility and response in various cancer types, including lung and liver cancers, further research should be directed at investigating their role in platinum-receiving urinary bladder cancer patients.

## Data Availability

Data sharing is not applicable to this article as no datasets were used during the current review.
